# Decreasing incidence of inflammatory bowel disease in Eastern Canada: a population database study

**DOI:** 10.1186/1471-230X-14-140

**Published:** 2014-08-09

**Authors:** Desmond Leddin, Hala Tamim, Adrian R Levy

**Affiliations:** 1 Department of Medicine, Dalhousie University, 912 Victoria, Victoria General Hsopital, Halifax, NS B3H 2Y9, Canada; 2 Epidemiology and Community Health, Dalhousie University, Halifax, Canada; 3 Kinesiology and Health Sciences, York University, Toronto, Canada

**Keywords:** Crohn’s, Inflammatory bowel disease, Ulcerative colitis, Epidemiology

## Abstract

**Background:**

Nova Scotia has one of the highest incidences of inflammatory bowel disease (IBD) in the world. We wished to determine trends of IBD over time.

**Methods:**

All Provincial residents have government provided health insurance and all interactions with the hospital, and physician billing systems, are captured on an administrative database. We used a validated measure to define incident cases of Crohn’s (CD), ulcerative colitis (UC) and undifferentiated IBD (IBDU). Incidence rates of these diseases for the years 1996–2009 were calculated.

**Results:**

Over the study period, 7,153 new cases of IBD were observed of which 3,046 cases were categorized as CD (42.6%), 2,960 as UC (41.4%) and 1,147 as IBDU (16.0%). Annual age standardized incidence rates were very high but have declined for CD from 27.4 to 17.7/100,000 population and for UC from 21.4 to 16.7/100,000. The decline was seen in all age groups and both genders. The decrease was not explained by a small increase in IBDU.

**Conclusion:**

The incidence of CD and UC are decreasing in Nova Scotia. If replicated elsewhere this indicates a reversal after a long period of increasing occurrence of IBD. This has implications for both epidemiology and health planning.

## Background

Inflammatory bowel disease (IBD) is a gastrointestinal disorder which consists of Crohn’s Disease (CD), Ulcerative Colitis (UC) and Undifferentiated IBD (IBDU). IBDU is diagnosed when the illness has features of both CD and UC [1].

The aetiology of these conditions remains unclear [2-6]. Incidence and prevalence rates are rising in many regions of the world including developing nations [2]. Escalating rates have led to an increased awareness of IBD given the significant burden of morbidity and high economic costs associated with it.

The prevalence and incidence of IBD has been studied extensively in recent years. The disease affects both genders and all ages. Molodecky et al. [3] conducted a comprehensive systematic review of the literature which compared CD and UC rates across regions and over time. The incidence and prevalence were highest in western nations, specifically Northern Europe and Canada.

In Canada studies have been conducted to describe patterns of IBD occurrence within this country. Bernstein et al. [7] analyzed data from five provinces and extrapolated to produce nationwide incidence and prevalence estimates for both CD and UC. Consistent with other studies, there were considerable differences in rates across regions. The age adjusted incidence of Crohn’s disease in Canada varied from 8.8/100,000 population in British Columbia to 20.2/100,000 in Nova Scotia.

The incidence of IBD varies with time [8,9]. Such changes may help identify factors contributing to the pathogenesis of the disease. Therefore it is important to measure a baseline and track changes over time using reliable and consistent data sources. The objective of this study was to determine the incidence of CD, UC and IBDU in Nova Scotia annually from 1996–2009 and assess changes in incidence over time.

## Methods

Nova Scotia is located on the east coast of Canada and has advantages as a location for the study of incidence trends in IBD. The population is ethnically homogenous, and there is little in- or out-migration [10]. The province has a population of just over 920,000 which is of sufficient size to yield enough cases to make meaningful inferences about changes in incidence, the population is equally divided between rural and urban areas, has a high background incidence and prevalence of the diseases [7], and it has universal health care in which all residents of the province receive necessary health care regardless of their ability to pay. All residents have a health card, which is used when accessing care. All encounters with the health system are captured on a government database, and it has a population health research unit, which has access to all health encounter data. This is not a registry study but is based on health utilization data which captures every resident of the Province.

### Data collection

The data for this study were based on the Nova Scotia administrative health care databases. All residents of the Province have publicly funded health insurance for physician and hospital care. Billings generated by patient interactions with hospital or physician services are submitted to the government for payment using unique patient identifiers. The Provincial Insurance payments database was used to determine Nova Scotia Medical Service Insurance eligibility, and for patient demographics. The Canadian Institute for Health Information Discharge Abstract database and Physician Billings claims database were used to obtain diagnosis and procedure codes for IBD. Records for the calendar years 1991 to 2010 inclusive were used.

The definition of IBD was based on a modification of the validated administrative case definition from Alberta [11] with a reported sensitivity and specificity of 77.9% and 99.8% respectively. Based on this case definition, Physician Claims and hospital Discharge Abstracts databases were searched for a diagnosis of IBD (i.e. ICD-9 codes of 555.x or 556.x; and ICD-10 codes of K50.x or K51.x).

Case definitions for CD (sensitivity of 91.1% and specificity of 98.7%) and UC (sensitivity of 81.7% and specificity of 97.4%) were also based on the validation study by Rezaie et al. [11] Within each administrative database (i.e. Discharge Abstract and Physician Billings claims databases) patients were given a +1 score for each ICD diagnostic code for UC (ICD-9 code 556.x, ICD-10 code K51.x) or a score of -1 for each ICD code for CD (ICD-10 code 555.x, ICD-10 code K50.x).

The diagnostic criteria are shown in Table 1. A patient with one hospitalisation or four physician claims would meet the criteria for a diagnosis of IBD. Following the diagnosis of IBD patients were sub classified by IBD type. If, for example, the patient had one hospitalisation with a discharge diagnosis of UC and one physician claims with a diagnosis of UC their cumulative score would be (+2 for the hospital discharge) + (+1 for the physician billing) = +3.

**Table 1:** The database criteria for the diagnosis of IBD, UC, CD and IBDU

**IBD**	All patients had to have at least one hospitalization, or four physician claims, within a two year period for a diagnosis of IBD.
• IBD patients were then classified as UC, CD or IBDU as follows.	
• Each medical contact for the Physician Billing claims database was scored as + 1 (UC) or -1 (CD).	
• Each medical contact for the Discharge Abstract database was scored as +2 (UC) or -2 (CD).	
• A cumulative score was calculated for each patient.	
**UC**	Patients with a cumulative score greater than +2.
**CD**	Patients with a cumulative score less than -2.
**IBDU**	Patients with a cumulative score equal to, or between, -2 and +2.

Since some subjects may not have been seen every year, incidence rates were calculated after excluding any subject with a physician billing or hospital discharge records with a diagnosis of the target diagnoses between 1991 and 1995. Hence, incidence rates were reported for the year 1996–2009. Date of first medical contact was considered the “diagnosis date” for estimating incidence rates.

### Statistical analysis

The total number of incident cases of CD, UC and IBDU were calculated for women and men and by age group. To adjust for age distribution and compare across years, directly age-standardized incidence rates of CD, UC and IBDU were calculated for women and men and overall. The 2006 Canadian population was used as the standard population. All rates were reported per 100,000 persons. Poisson regression was used to measure the effect of calendar year on the incidence of CD, UC, IBDU and IBD for each sex by age groups and overall. Year was included in the model as a categorical variable with the year 1996 being the reference category. Analysis was conducted using SAS 9.1 software [12].

### Ethics approval

This study was approved by the Dalhousie University ethics committee and by the Capital District Health Authority ethics committee and is in compliance with the Helsinki Declaration. Access to the health databases is controlled by Health Data Nova Scotia, and was approved by the access committee following review of the application and receipt of ethics approval.

## Results and discussion

Over the 14 year period under study, across all age groups and both genders, a total of 7,153 incident cases of IBD were identified with 3,046 cases categorized as CD (42.6%), 2,960 cases categorized as UC (41.4%) and 1,147 cases categorized as IBDU (16.0%).

With regard to the 3,046 incident cases of CD, 1,743 (57.2%) were in women and 1,303 (42.8%) were in men whereas of the 2,960 incident cases of UC, 1,507 (50.9%) were in women and 1,453 (49.1%) in men. Of the 1,147 incident cases of IBDU, 617 (53.8%) were in women and 530 (46.2%) in men. Over 50% of the incident cases of IBD in men and women occurred among ages 20–59 years (Table 2).

**Table 2:** Total number (%) of incident cases of CD, UC and IBDU in Nova Scotia, 1996-2009

	**CD**		**UC**		**IBDU**	
**Age group (years)**	**N**	**%**	**N**	**%**	**N**	**%**
**Women**						
<10	23	1.3	12	0.8	4	0.6
10-19	193	11.1	102	6.8	20	3.2
20-29	397	22.8	212	14.1	76	12.3
30-39	364	20.9	316	21.0	103	16.7
40-49	267	15.3	309	20.5	97	15.7
50-59	237	13.6	244	16.2	101	16.4
60-69	148	8.5	168	11.1	80	13.0
70-79	83	4.8	94	6.2	82	13.3
≥ 80	31	1.8	50	3.3	54	8.8
All women	1743	100.0	1507	100.0	617	100.0
**Men**						
<10	25	1.9	14	1.0	2	0.4
10-19	221	17.0	97	6.7	27	5.1
20-29	285	21.9	183	12.6	74	14.0
30-39	251	19.3	252	17.3	85	16.0
40-49	218	16.7	301	20.7	78	14.7
50-59	156	12.0	265	18.2	106	20.0
60-69	92	7.1	194	13.4	77	14.5
	**CD**		**UC**		**IBDU**	
**Age group (years)**	**N**	**%**	**N**	**%**	**N**	**%**
70-79	43	3.3	112	7.7	57	10.8
≥ 80	12	0.9	35	2.4	24	4.5
All men	1303	100.0	1453	100.0	530	100.0

*Abbreviations:CD* Crohn’s disease, *IBDU* Undifferentiated inflammatory bowel disease, *UC* Ulcerative colitis.

**Figure 1:**
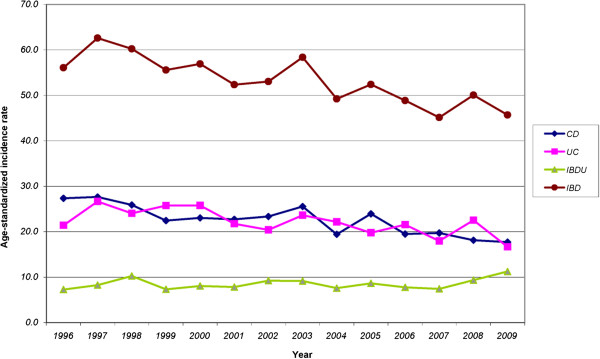
Age-standardized incidence rates of Crohn’s Disease (CD), Ulcerative Colitis (UC), Undifferentiated Inflammatory Bowel Disease (IBDU) and Inflammatory Bowel Disease (IBD) in Nova Scotia, 1996–2009.

The directly age-standardized rates of CD, UC and IBDU allowed for comparisons across years with adjustment for age distribution over time. Decreases in incidence were observed both for women (CD from 31.5 to 19.1/100,000 and UC from 20.3 to 16.9/100,000) and men (CD 23.0 to 16.3 and UC from 22.6 to 16.6 per 100,000) over the study period. Age-standardized rates of IBDU increased both for women (from 8.4 to 12.1) and men (from 6.2 to 10.4) over study period (Table 3 and Figure 1).

**Table 3:** Annual directly age-standardized incidence rates per 100,000 of CD, UC and IBDU by sex in Nova Scotia, 1996-2009

	**Women**			**Men**			**Sexes combined**		
	**CD**	**UC**	**IBDU**	**CD**	**UC**	**IBDU**	**CD**	**UC**	**IBDU**
**Year**									
1996	31.5	20.3	8.4	23.0	22.6	6.2	27.4	21.4	7.3
1997	29.1	25.2	8.8	26.1	28.2	7.7	27.7	26.7	8.3
1998	30.2	22.1	11.5	21.3	26.1	9.1	25.9	24.1	10.3
1999	24.5	26.1	6.8	20.3	25.4	7.9	22.5	25.8	7.4
2000	28.2	23.9	8.1	17.6	27.8	8.0	23.1	25.8	8.1
2001	22.2	21.5	9.4	23.4	22.0	6.2	22.8	21.8	7.9
2002	25.2	20.9	9.0	21.5	20.0	9.5	23.4	20.4	9.3
2003	28.6	24.2	10.0	22.4	23.1	8.4	25.6	23.6	9.2
2004	22.6	23.1	6.8	16.2	21.3	8.5	19.4	22.2	7.6
2005	27.0	22.2	8.8	20.7	17.2	8.6	24.0	19.8	8.7
2006	23.2	21.3	7.5	15.7	21.8	8.1	19.5	21.6	7.8
2007	21.2	19.1	7.6	18.1	16.8	7.2	19.7	18.0	7.4
2008	21.1	19.8	10.4	15.1	25.3	8.4	18.2	22.5	9.4
2009	19.1	16.9	12.1	16.3	16.6	10.4	17.7	16.7	11.3

*Abbreviations*: *CD* Crohn’s disease, *IBDU* Undifferentiated inflammatory bowel disease, *UC* Ulcerative colitis.

The results in Table 3 are not based on the regression model. It is descriptive and we cannot make inference as to whether there is significant difference in incidence over time. The percent differences in incidence rate with time that is calculated from Table 4 are based on the Poisson regression where the outcome follows a Poisson distribution.

**Table 4:** Estimated age-sex specific percentage changes (95% confidence intervals) in rates of CD, UC, IBDU and IBD in Nova Scotia, 1996-2009

	**CD**	**UC**	**IBDU**	**IBD**
	**% change (95%CI)**	**% change (95%CI)**	**% change (95%CI)**	**% change (95%CI)**
**Women**				
<20	-25.7 (-69.7, 82)	40.1 (-59, 378)	48 (-91.5, 2487)	-0.7 (-50.5, 99.3)
20-29	-45.8 (-77.9, 33)	-37.8 (-81.7, 111.5)	20.9 (-84.1, 819.2)	-38.1 (-68.7, 22.3)
30-39	-29.6 (-71.7, 75.3)	-35.3 (-75.8, 72.9)	-18.9 (-85.3, 347.7)	-30.7 (-62.8, 29.2)
40-49	-16.9 (-68.3, 117.8)	-11.3 (-63.8, 117.1)	23.7 (-75.1, 515.1)	-9.3 (-50.6, 66.3)
50+	-28.5 (-55.9, 16)	-14.2 (-45.8, 35.7)	8.7 (-40.9, 100)	-15.2 (-36.7, 13.5)
All women (adjusted for age)	-31.9 (-41.6, -20.7)	-20 (-32.5, -5.3)	11.5 (-15.2, 46.6)	-21.4 (-29, -13)
**Men**				
<20	-19.9 (-64.1, 78.6)	111.7 (-36.4, 603.9)	126.6 (-82, 2753)	17.1 (-38.1, 121.5)
20-29	-46.9 (-81.2, 50.6)	-43.4 (-84.5, 107.2)	78.4 (-76.8, 1269.4)	-35.8 (-69.7, 35.9)
30-39	-27 (-75.6, 118.2)	-15.5 (-71.6, 151.3)	31.3 (-79.8, 751.4)	-15.4 (-58.5, 72.7)
	**CD**	**UC**	**IBDU**	**IBD**
	**% change (95%CI)**	**% change (95%CI)**	**% change (95%CI)**	**% change (95%CI)**
40-49	-37 (-78.2, 81.9)	-46.8 (-78.5, 31.8)	68.6 (-71.9, 910.5)	-34.2 (-65.3, 24.9)
50+	-20.1 (-58.2, 52.9)	-32.7 (-57.4, 6.5)	-11.3 (-55.8, 77.8)	-25.1 (-46.2, 4.1)
All men (adjusted for age)	-32.9 (-43.8, -19.9)	-29 (-40.3, -15.6)	21.2 (-9.5, 62.4)	-24.3 (-32.2, -15.5)
**Sexes combined**				
<20	-22.6 (-57.5, 40.8)	71.4 (-27.3, 304)	108.3 (-69.2, 1307)	8.2 (-32.4, 73.3)
20-29	-46.4 (-72.8, 5.8)	-40.5 (-75.5, 44.9)	46.3 (-65.2, 515.2)	-37.2 (-62, 4)
30-39	-28.8 (-64.7, 43.6)	-27.2 (-64.9, 51)	0.8 (-71.4, 254.9)	-24.6 (-52.8, 20.5)
40-49	-26.6 (-64, 49.7)	-31 (-63.5, 30.2)	42 (-57, 368.4)	-22 (-49.8, 21.1)
50+	-25.8 (-49.7, 9.6)	-24.2 (-45.1, 4.8)	-1 (-37.4, 56.7)	-20 (-35.7, -0.4)
Sexes combined (adjusted for age)	-32.5 (-39.8, -24.2)	-24.8 (-33.2, -15.3)	17 (-4, 42.6)	-22.8 (-28.4, -16.8)

*Abbreviations*: *CI* Confidence Interval, *CD* Crohn’s disease, *IBD* Inflammatory bowel disease, *IBDU* Undifferentiated inflammatory bowel disease *UC* Ulcerative colitis.

**Figure 2:**
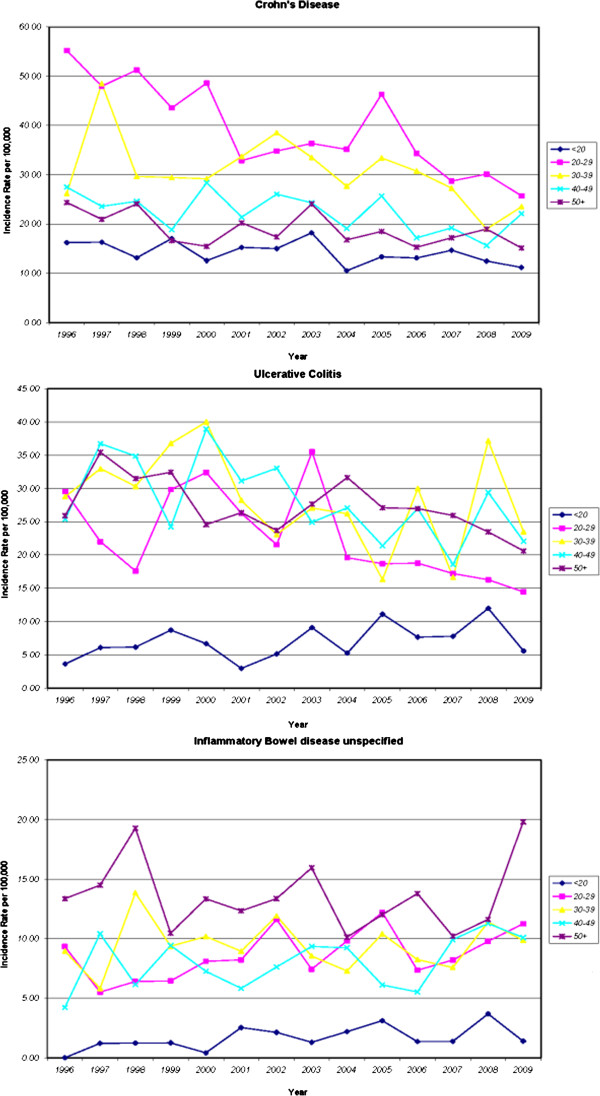
Annual incidence rates per 100,000 of Crohn’s Disease (CD), Ulcerative Colitis (UC) and Undifferentiated Inflammatory Bowel Disease (IBDU) by Age, Nova Scotia, 1996–2009.

**Figure 3:**
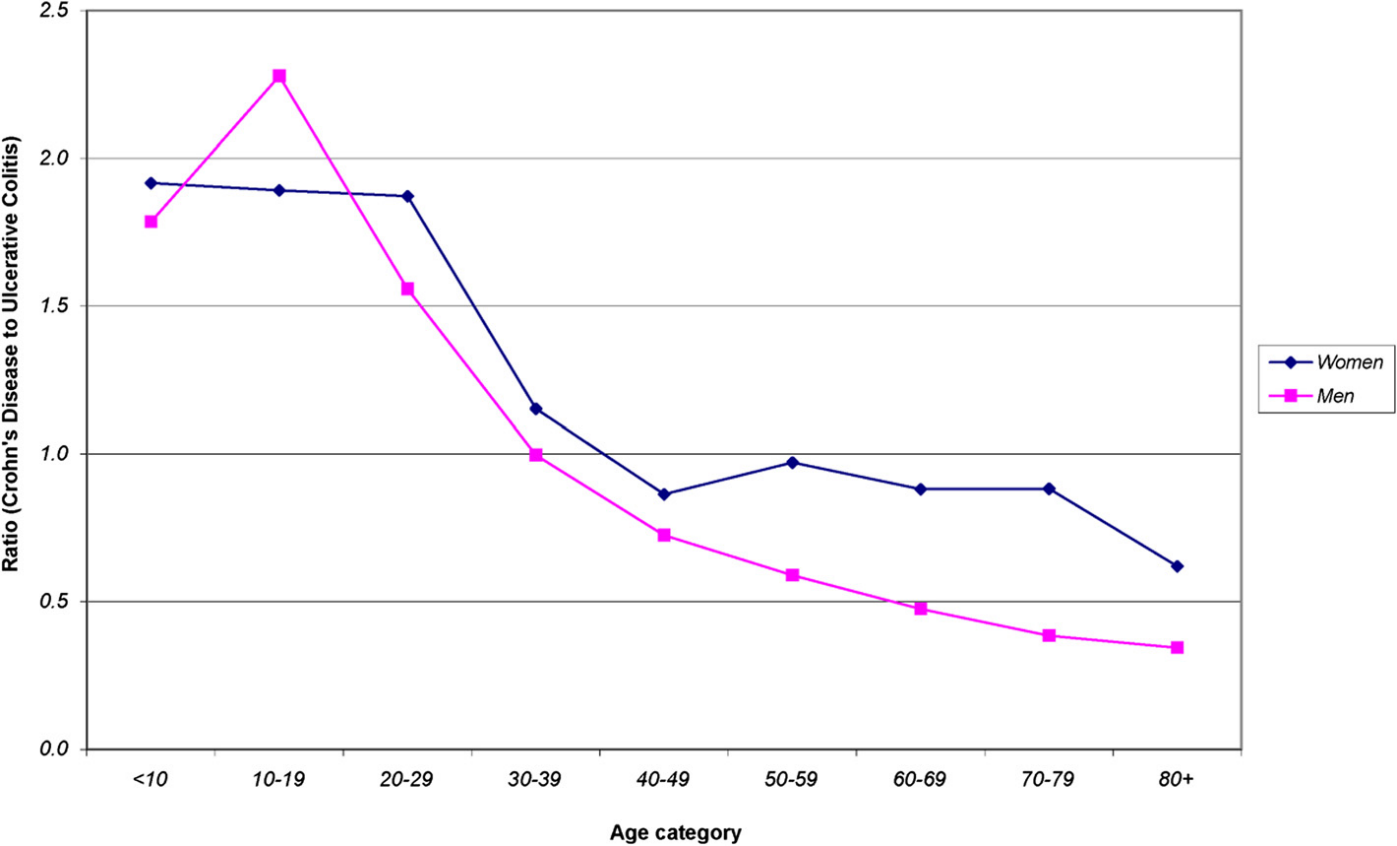
Ratio of incidence of Crohn’s Disease (CD) to Ulcerative Colitis (UC) by Age in Nova Scotia, 1996–2009.

Results of the Poisson regression showed that there was a significant negative change in the overall age-adjusted rates relative to 1996 for CD and UC (Table 4). Among sexes combined, the decrease for CD was 32.5% (95% CI, -39.8 to -24.2) and for UC 24.8% (95% CI, -33.2 to -15.3). In men, the decrease for CD was 32.9% (95% CI, -43.8 to -19.9) and for UC 29.0% (95% CI, -40.3 to -15.6). In women, the decrease for CD was 31.9% (95% CI, -41.6 to -20.7) and 20.0% (95% CI, -32.5 to -5.3) for UC.Figure 2 shows incidence rates for CD, UC and IBDU over time for different age groups. For CD, a decrease in incidence rates over time was observed for all age groups. For UC, a decrease in incidence rates was observed for all age groups except for age group less than 20 years of age where incidence rates increased over time period. On the other hand, incidence rates of IBDU showed no consistent change for different age groups over time.Incidence rates of CD among youngest women in our cohort were almost double the rates of UC. This trend appears to level off for women 30 years of age and older where rates of CD and UC became equal. The same trend was not observed for men, where rates of CD were higher than UC for those younger than 30 years of age. The equalization of the ratio of CD Disease to UC occurred at age of 30–39 years, at which point the ratio between CD and UC reversed (Figure 3).

Using a proxy for the incidence of both CD and UC we have shown evidence of a decrease in incidence of approximately 32% and 25%, respectively, over a fourteen-year period from 1996–2009. The decline was observed in all age groups, for both diseases, and both genders with the exception of a non-significant increasing trend in ulcerative colitis under the age of 20.

Results of the present study showed that age-standardized incidence rates of CD and UC for NS for the years 1998–2000 were higher than those reported by Bernstein et al. [7]. This is explained by differences in the case definitions used for each of the studies. In Bernstein et al. the IBD case definition required a patient to have at least five physician claims or hospitalizations for CD or UC if resident of the province for at least two years (or at least three claims if resident of the province for fewer than two years). In contrast, the definition of IBD used here, based on Rezaie et al. [11], relied on at least one hospitalization or four physician claims with an IBD diagnostic code within a 2-year period. Bernstein et al. [5] note that their definition leads to an underestimate of the incidence of IBD. Fransoo et al. [13] make clear that the Bernstein et al. case definition is better for estimating population prevalence than incidence of IBD. In addition the definition of Rezaie et al. [11] but not that of Bernstein et al. [5] captures cases best defined as IBDU. Our definition of IBD may underestimate milder cases who are not seen frequently, and therefore do not generate billing data, or who are not hospitalized.

The possibility of information bias is a concern. We mitigated this by using a validated measure of health care access to define incident cases [11]. We incorporated a wash out period of 5 years, to reduce the impact of including prevalent cases. Most importantly this is a comprehensive population based study. Improvements in treatment may have led to a decrease in interactions resulting in fewer patients meeting the definition. The major change in management has been the introduction of the biologics. Patients who are being treated with biologics are seen regularly in follow up. We also considered whether our data simply shows regression to the mean and consider this unlikely given the duration of the decline extending, as it does, over a period of fourteen years. The results could be secondary to reduced access to care, or a reduction in colonoscopy. Data from the Nova Scotia Department of Health (personal communication) shows that the number of colonoscopies in the Province increased by nearly 300% over the years of the study. This would favour an increase in IBD diagnosis, and IBD related billings, rather than the decrease observed.

There is no universally agreed national or international standard definition of IBD which can be utilised for IBD data base studies. A national study is required using a standardized case definition to verify our results.

The numbers of new cases of CD and UC were almost equal in our population but CD was more common in younger age groups. The incidence CD and UC equalize at age 50 years for women but for men over the age of 50 years UC was more common than CD. This is consistent with the prevalence of these diseases observed in a US population [14].

These results are in contrast to those reported in a recent systematic review (3), which concluded that no studies of CD disease, and only 6% of studies of UC, have shown a significant decline in incidence. However, there are few population-based studies of incidence over time, and none of a population with a baseline incidence comparable to ours. Even fewer studies have reported incidence for the years beyond 2003. As can be seen from Table 3 a small decline in incidence was evident by 2003 but is more obvious from 2003–2009.

While no studies have reported a significant decline in CD several have shown either a small, non-significant decrease [15-18] or a plateau of incidence [19]. The report from Northern France, which described the incidence up to 2007, showed that the incidence of CD peaked in 1999 and is trending slightly downwards since [19].

The incidence for ulcerative colitis in the younger age groups showed a non significant trend upwards over the period under study. The incidence of IBD in the paediatric population has been reviewed [20]. Sixty percent of studies reported an increasing incidence of CD and 20% of studies reported a rise in UC. The results reported here are consistent with those findings. It is possible that genetic factors play a greater role in younger onset IBD whereas environmental factors may be of greater importance in older age onset disease.

We observed a small and non significant increase in IBDU. Clinically IBDU is diagnosed when the disease has features of both ulcerative colitis and Crohn’s disease [1]. Clinically IBDU may account for up to 12.6% of new cases of IBD in children and from 6% to 11.5% of cases in adults [21,22], a proportion similar to that observed in our study. However we used a database scoring system to define cases of IBDU, not a clinical definition, and this may account for the relatively high percentage of IBDU cases in this study. It is possible that some of these cases are actually UC or CD but even if they were ascribed to either group the total number of new cases of IBD shows a decline.

There is little in- or out-migration to Nova Scotia therefore the genetic pool is stable [10] and the results may point to an environmental factor. One environmental change, with the potential to affect all residents, was the addition of folic acid to cereals starting in 1998. This was done to reduce the incidence of neural tube defects in newborns [23].

## Conclusions

The evidence from this study indicates that incidence rates of both CD and UC are declining. Similar studies of secular trends in these conditions from other jurisdictions will be of interest to determine if the decline is a more universal observation. These results need to be replicated in countries with a high incidence and they may only to applicable to areas where the starting incidence is high. If replicated elsewhere this indicates a reversal after a long period of increasing occurrence of IBD. This has implications for both epidemiology and health planning.

## Abbreviations

CD: Crohn’s disease; IBDU: Undifferentiated inflammatory bowel disease; UC: Ulcerative colitis.

## Competing interests

The authors declare that they have no competing interests.

## Authors’ contributions

DL developed the project and conceived the idea behind the study and was involved in interpretation and writing the manuscript. HT was involved in the design, execution of the study, analysis and writing. AL provided intellectual input, guidance and directly contributed to interpretation. All authors read and approved the final manuscript.

## Pre-publication history

The pre-publication history for this paper can be accessed here:

http://www.biomedcentral.com/1471-230X/14/140/prepub
